# Circular RNAs in Cardiovascular Disease: An Overview

**DOI:** 10.1155/2017/5135781

**Published:** 2017-01-22

**Authors:** Ximin Fan, Xinyu Weng, Yifan Zhao, Wei Chen, Tianyi Gan, Dachun Xu

**Affiliations:** ^1^Department of Cardiology, Shanghai Tenth People's Hospital, Tongji University School of Medicine, Shanghai, China; ^2^State Key Laboratory of Cardiovascular Disease, Heart Failure Center Fuwai Hospital, National Center for Cardiovascular Diseases, Chinese Academy of Medical Sciences and Peking Union Medical College, Beijing, China

## Abstract

Circular RNA (circRNA), a novel type of endogenous noncoding RNA (ncRNA), has become a research hotspot in recent years. CircRNAs are abundant and stably exist in creatures, and they are found with covalently closed loop structures in which they are quite different from linear RNAs. Nowadays, an increasing number of scientists have demonstrated that circRNAs may have played an essential role in the regulation of gene expression, especially acting as miRNA sponges, and have described the potential mechanisms of several circRNAs in diseases, hinting at their clinical therapeutic values. In this review, the authors summarized the current understandings of the biogenesis and properties of circRNAs and their functions and role as biomarkers in cardiovascular diseases.

## 1. Introduction

Circular RNA (circRNA) has been recently discovered and is becoming a research focus in the field of untranslated RNA. Circular RNAs are a kind of competing endogenous RNAs (ceRNA) [1] which can regulate RNAs. Currently, there are two types of ceRNAs including coding and noncoding RNAs. The latter includes long noncoding RNAs (lncRNA), microRNAs (miRNA), and circular RNAs (circRNA) [2]. CircRNAs are predominantly found in cytoplasm and are highly stable in human bodies. They are also identified in various organisms [3–6] and are abundantly expressed and evolutionarily conserved across the eukaryotic tree of life [7, 8], especially in human and mice [7]. In this review, we have summarized the current studies of the biogenesis, the properties and the functions of circRNAs, and their roles in cardiovascular diseases.

## 2. Biogenesis of CircRNAs

**Figure 1:**
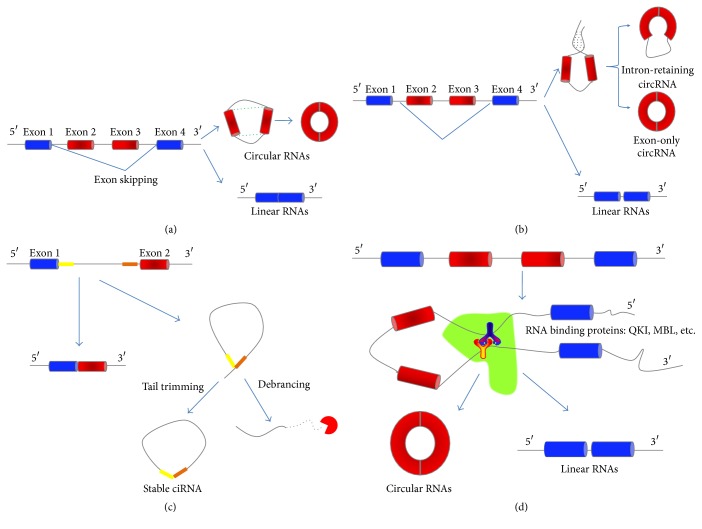
Models of circRNA biogenesis: (a) lariat-driven circularization: exon skipping leads to a covalent splice of the splice donor in 3′ end of exon 1 to the splice acceptor in 5′ end of exon 4, which forms a linear product and a lariat structure containing the skipped exons 2 and 3. Then the lariat is joined by spliceosome and the introns are removed to form a circRNA. (b) Intron-pairing-driven circularization: intron 1 and intron 3, which contain complementary sequence motifs, lead to close proximity through direct base-pairing and form a linear RNA and a circular structure. The splicing of the two introns produces a circRNA (exon-only circRNA), while the generation of an EIciRNA (intron-retaining circRNA) is caused by the presence of a retained intron. (c) Circular intronic RNAs: the existence of 7 nt GU-rich element near exon 1 (yellow box) and 11 nt C-rich element near exon 2 (orange box) makes it possible for an intron to escape debranching when the intron lariat is produced from the splicing reaction. (d) RNA binding proteins (RBPs) driven circularization: the interaction between RBPs (Y-shape) can bind to sequence motifs and bring two flanking introns close together. Then the introns are removed to form a circRNA.

CircRNAs mainly come from the exons of protein-coding genes, and they are not supposed to be produced by a normal mode of RNA splicing [9]. They differ structurally from other RNAs in that they are circularized by joining the 3′ and 5′ ends together via exon circularization or intron circularization [10]. Jeck and his colleagues have proposed two different models of exon circularization of circRNA: “lariat-driven circularization” (Figure 1(a)) and “intron-pairing-driven circularization” (Figure 1(b)) [11]. The former is associated with exon skipping, in which one or more exons of the transcript are skipped, and it contributes to an exon-containing lariat. The lariat itself would then be joined by spliceosome and become an exon circle. The latter is mostly related to complementary motifs, which are present in the intronic regions. Accumulated evidence has verified that pairing between these motifs induces circularization [11]. The genome-wide analyses observed that complementary flanking Alu elements are important for the formation of circRNAs [12]. However, Alu elements are not specifically needed if there are other inverted repeats [13]. That is to say circRNA can also be generated via direct base-pairing in the presence of inverted repeat without Alu elements. Nevertheless, more than half of the known circRNAs do not have complementary flanking in the intronic sequences, as it has been reported that 38% of all known* Caenorhabditis elegans *circRNAs and 9% of all known human circRNAs can be recognized using the base-pairing potential to cyclize the exons [14]. In these cases, their introns were found to be remarkably longer than average [15, 16]. After two introns forming circular structure via base-pairing, the two introns are removed or retained to form a circRNA (exon-only circRNA) or an EIciRNA (intron-retaining circRNA) [11, 17]. The intron-retaining circRNAs were found recently by Li et al. who termed them exon-intron circRNAs or EIciRNAs [17]. However, the mechanism of their formation is not clear yet. Soon after Jeck, Zhang et al. proposed a model of circular intronic RNAs (ciRNAs), suggesting that circRNAs could also come from lariat introns that escape debranching. The generation of ciRNA is related to a 7 nt GU-rich element near the 5′ splicing site and an 11 nt C-rich element near the branching point (Figure 1(c)) [18]. Judging from the above findings, circRNAs can also arise from introns. Recent studies have also suggested another pathway of circRNA biogenesis through RNA binding proteins (RBPs). Quaking protein (QKI) and Muscleblind protein (MBL) can bind certain circRNA flanking introns and act as RBPs to bring two flanking intronic sequences close together, hence provoking the circularization (Figure 1(d)) [19, 20].

## 3. Properties of CircRNAs

There are several important properties of circRNAs. First, they are widely expressed in human cells, and their expression levels are more than 10 times higher than those of the corresponding linear mRNAs [9, 11]. Second, different from traditional linear RNAs, circRNAs form covalently closed loop structures with neither 5′-3′ polarities nor polyadenylated tails, resulting in less degradation by RNA exonuclease or RNase R, which makes them more stable than linear RNAs in human bodies [21, 22]. Third, besides a few circRNAs, most of them have highly conserved sequences between species [9, 11, 12, 22]. Fourth, the vast majority of them reside in cytoplasm [23], while only a small portion is in cell nucleus [12]. Fifth, they primarily come from exons, while a few others come from introns or intron fragments. Sixth, some circRNAs have microRNA response element (MRE), through which they can have interactions with miRNAs, thus regulating target gene expressions [13, 24]. Seventh, most circRNAs are endogenous noncoding RNAs (ncRNA) [9]. Eighth, the majority of circRNAs can play regulatory roles in transcription and posttranscription, and only a few play roles in transcription [18]. Ninth, similar to linear mRNAs, circRNAs also show tissue-specific and/or developmental-stage-specific expression [8, 25].

## 4. Function of CircRNAs

As circRNAs are abundant and evolutionarily conserved, several potential functions of circRNAs have been predicted [10, 26]. Some studies have revealed that circRNAs could function as microRNA (miRNA) sponges, modulate alternative splicing or transcription, and regulate the expression of parental genes [8, 13, 17, 18, 20], among which the function as miRNA sponges has been a focus of recent research.

### 4.1. CircRNAs Function as MiRNA Sponges

CircRNAs are a kind of competing endogenous RNAs (ceRNA), which contain shared miRNA response elements (MRE) [27]. Therefore, the presence or absence of circRNAs would affect the activities of miRNAs. CircRNAs can competitively bind to miRNAs, which results in the reduction of miRNA molecules. Then the reduced miRNAs would have less inhibiting effects on miRNA target genes, resulting in the upregulation of the expression of miRNA target genes [8]. As circRNAs have many miRNA binding sites and can absorb miRNAs like a sponge, they are initially demonstrated as miRNA sponges [8, 13].

The most representative circRNA is Cdr1as (antisense to the cerebellar degeneration-related protein 1 transcript) [28], also termed as ciRS-7 (circular RNA sponge for miR-7), and Cdr1as has a negative regulation of the activity of miR-7 [13]. According to the bioinformatics, Cdr1as contains more than 70 binding sites for miR-7 and is bound by Argonaute proteins and functions as a miR-7 sponge in brain tissues [13], islet cells [29], and some other cells. The expressions of published miR-7 target genes [8, 13], such as SNCA, EGFR, and IRS2 [30–32], are downregulated by silencing of Cdr2as [13]. Some other studies also demonstrated that miR-7 can regulate the occurrence of various cancers through repressing its target genes like EGFR (epidermal growth factor receptor) [33], ACK1 (Activated Cdc42-associated Tyrosine Kinase 1) [34], or IGF1R (insulin-like growth factor 1 receptor) [35]. Similar to Cdr1as, murine sex-determining region Y (Sry) is another recognized circRNA with 16 biding sites for miR-138 and is likely to act as a miR-138 sponge [13]. Hence, the findings that circRNAs can interact with miRNAs and alter the expression of miRNAs and/or its target genes [8, 13] may have great significance in the pathogenesis research for related diseases and may point out a potential direction of future treatment as well.

### 4.2. CircRNAs Modulate Alternative Splicing or Transcription

**Figure 2:**
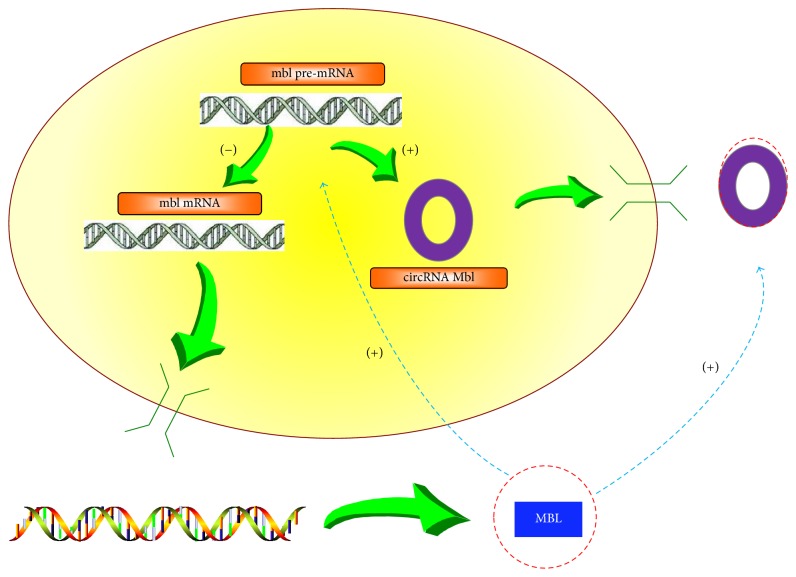
A model of the generation of circular Mbl (circMbl) and MBL: CircMbl is derived from the mbl locus and has conserved binding sites for the MBL protein in the flanking intronic sequences, so it can bind MBL protein. Besides, BML levels can strongly affect the splicing process when mbl pre-mRNA produces mbl mRNA and circMbl. When the level of MBL is high, mbl pre-mRNA produces less mbl mRNA and more circMbl. Less mbl mRNA generates less MBL and more circMbl binds to more MBL, so that MBL level decreases. (+) represents promotion while (−) represents inhibition in this figure, and vice versa.

MBL protein is a splicing factor which can affect alternative mbl pre-mRNA splicing during generation of mbl mRNA and circular Mbl (circMbl). CircMbl derived from the* mbl* locus has conserved binding sites for the MBL protein in the flanking intronic sequences. Reut Ashwal-Fluss has verified that MBL levels can strongly affect circMbl biosynthesis when MBL proteins bind to both introns of the circMbl simultaneously, rather than the large total number of MBL binding sites on the circMbl alone. Moreover, a balance between circMbl biogenesis and splicing has been proposed. When MBL is in excess, mbl pre-mRNA will produce more circMbl and less mbl mRNA, resulting in the decrease of MBL levels, because more circMbl binds to more MBL while less mRNA produces less MBL (Figure 2) [20]. These findings may hint that some circRNA may play a role in controlling the expression of mRNA through binding to RNA binding proteins (RBPs) and affecting the canonical splicing.

As stated above, the three types of circRNAs, namely, ciRNA, circRNA, and EIciRNA, resulted from the competition of complementary flanking in or across the intronic sequences. For those circRNAs and the corresponding linear mRNAs which are generated from a single gene locus, their progressions are closely related, as complementary intronic sequences can lead to more linear mRNA generation, while complementary sequences flanking across the intronic sequences can promote exon circularization. Therefore, circRNAs are interrelated with each other, and the competitive balance among them can affect the expression of mRNA [12, 36].

### 4.3. CircRNAs Regulate Parental Gene Expression

**Figure 3:**
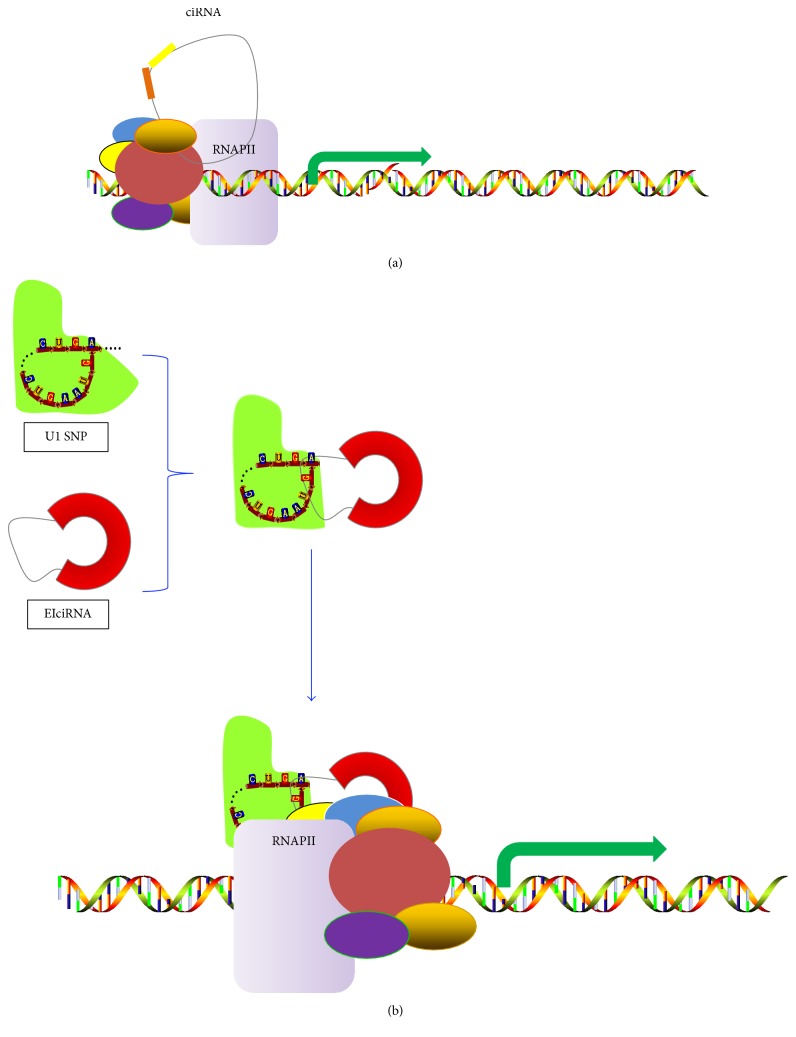
Model of circRNA regulating the expression of a parental gene: (a) CiRNAs come from lariat introns that escape debranching. CiRNA can bind to RNA polymerase II (RNA pol II) and function in the promoter region of genes. (b) EIciRNA can interact with the U1 snRNP (small nuclear ribonucleoprotein) via specific RNA-RNA interaction between U1 snRNP and EIciRNA to form EIciRNA-U1 snRNP complexes, and the complexes will recruit RNA pol II to the promoter to stimulate host gene expression.

EIciRNAs and ciRNAs were recently revealed to promote transcription of the parental genes. Zhang and others found that ciRNAs have few microRNA binding sites, suggesting that they may function differently [37]. Some ciRNAs are predominantly localized in the nucleus and can promote host-gene transcription through interacting with the RNA polymerase II (pol II) in the promoter region of genes (Figure 3(a)) [18, 38]. EIciRNAs are also abundant in nucleus, such as circEIF3J and circPAIP2, and can bind to U1 snRNP (small nuclear ribonucleoproteins) in nucleus to form EIciRNA-U1 snRNP complexes. The complexes can interact with RNA polymerase II in the promoter region of genes, therefore enhancing the transcription of their parental genes (Figure 3(b)) [17, 38]. In some cases, when both circRNA and 3′-untranslated region (UTR) of the transcript from their parental gene share the same miRNA binding sites, circRNA can act as miRNA sponge in the cytoplasm to improve the translations of the transcript from their parental gene. Li and his colleagues found that cir-ITCH share the same miRNA (miR-7, miR-17, and miR-214) binding sites with 3′-UTR of ITCH mRNA and increase ITCH protein levels [39]. Here, we speculate that intronic circRNAs may be efficient for regulatory functions in the cell nucleus close to the transcription site, while the generalized exon-only circRNAs may fulfill transcriptional regulation in cytoplasm [36].

## 5. Roles in Cardiovascular Diseases

Several recent studies have suggested that circRNAs may play essential roles in the initiation and development of cardiovascular diseases (Table 1).

### 5.1. Circular RNA HRCR Protects Heart from Pathological Hypertrophy and Heart Failure

MiR-223 is an endogenous regulator which can induce cardiac hypertrophy and heart failure [44], as well as hypertrophy in cardiomyocytes [45]. ARC (apoptosis repressor with CARD domain) is a downstream target of miR-223 [46], indicating that miR-223 exerts its effect through ARC. It has been reported that ARC play a protective role in cardiomyocyte hypertrophy and apoptosis [47–49]. As circRNAs act as miRNA sponges to interact with miRNAs and influence the expression of miRNA [8, 13], Wang and his team proposed and verified that the heart-related circRNA (HRCR) can directly bind to miR-223 and act as an endogenous miR-223 sponge to inhibit miR-223 activity, which results in the increase of ARC expression [46]. Taken together, HRCR acts as an endogenous miR-223 sponge to modulate the expression of miR-223 and ARC, through which it regulates cardiomyocytes hypertrophy as well as cardiac hypertrophy and heart failure. Thus, it is speculated that enforced expression of HRCR attenuates the development of cardiac hypertrophy and heart failure. Furthermore, it may be targeted for drug development in the inhibition of related cardiovascular disorders.

### 5.2. Circular RNA Cdr1as Induces Myocardial Infarction

Myocardial infarction (MI) has been one of the leading causes of death and disability around the world [50]. During MI development, prolonged myocardial ischemia contributes to the myocardial cell death process [51], namely, apoptosis, one programmed cell death.

Recently, one of the circRNAs, Cdr1as, was described to function as miR-7 sponges and can inhibit the activity of miR-7 [8, 13]. Zhao and his colleagues proposed that miR-7a/b can act as protective roles in myocardial cells through negatively regulating PARP and decreasing apoptosis [52]. SP1 and PARP (poly ADP-ribose polymerase) are miR-7 target genes [53] and can inhibit miR-7a-induced decrease of cell apoptosis under hypoxia treatment [53] and play proapoptotic roles during MI development [54, 55]. Therefore, Cdr1as can function as a miR-7a sponge in promoting MI injuries through reducing the activity of miR-7a and upgrading the expression of miR-7a targets like PARP and SP1, which indicates the key role of Cdr1as/miR-7a axle in MI-induced myocardial apoptosis.

In terms of this new finding that Cdr1as functions as a miR-7a sponge in myocardial cells and regulates miR-7a, PARP, and SP1 in MI injury, further efforts can be done to move forward and figure out the potential therapeutic value for improving MI-related injuries.

### 5.3. Circular RNA Circ-Foxo3 Promotes Cardiac Senescence

Circ-Foxo3 is derived from a member of the forkhead family of transcription factors which is called Foxo3 and has been detected to be highly expressed in the cytoplasm of aged mice and patients [56]. A number of researches have proved that the high expressions of circ-Foxo3 accompanied with more cells were held up at G1 phase and unable to transit to S phase. Based on the previous findings, Du et al. proposed that the expression of the circ-Foxo3 represses cell proliferation and cell cycle progression [57].

Transcription factors ID1, E2F1, FAK, and HIF1a play an antisenescence role while entering cell nucleus, but circ-Foxo3 could repress this antiageing effect by hindering their transfer to nucleus. The gain-and-loss of function experiment also demonstrated a positive relation between circ-Foxp3 and senescence [56].

Therefore, the ectopic expression of circ-Foxo3 is supposed to promote senescence through arresting and relocating ID1, E2F1, FAK, and HIF1a in cytoplasm and blocking their antisenescent function. These findings may provide new insights into the inhibition of cardiac senescence and myocardial protection.

### 5.4. Circular RNA cANRIL Is Correlated with Atherosclerosis Risk

Several genome-wide association studies (GWAS) have revealed the links between single nucleotide polymorphisms (SNPs) on chromosome 9p21 near the* INK4/ARF* locus and ASVD [58, 59], suggesting that chromosome 9p21.3 is correlated to the susceptibility of ASVD. Liu and his colleagues verified that the ASVD-associated SNPs at 9p21 can control INK4/ARF (cyclin-dependent kinase 4 inhibitor, INK4a; alternative reading frame, ARF) expression [60]. Circular ANRIL RNA (circular antisense noncoding RNA in the* INK4* locus, cANRIL) is an antisense transcript from the* INK4A/ARF* locus [61]. SNPs on chromosome 9p21.3 near the* INK4/ARF* locus regulate INK4/ARF transcription through modulating ANRIL splicing and cANRIL production [40], indicating that the structure and expression of cANRIL species resulted from the alternative splicing in the process of INK4/ARF transcription. Jacobs and colleagues figured out that the* INK4/ARF* locus can be inhibited by Polycomb group (PcG) complexes [62]. As cANRIL can influence the PcG-mediated INK4/ARF silencing through recruiting PcG complexes [63–65], Burd and his colleagues speculated that modified cANRIL structure can result in changes in PcG-mediated INK4/ARF silencing and atherosclerosis susceptibility [40]. They also suggested that cANRIL expression may be a useful marker which is of pathogenic relevance to ASVD susceptibility [40].

## 6. Conclusion

CircRNAs are now a noticeable area in the field of RNA. It is clear that natural circRNAs are an abundant, diverse, stable, and conserved class of RNA molecules, representing a new type of regulatory noncoding RNA. Nevertheless, their biological roles are not yet clearly understood, as well as their localization and degradation. Recent researches have demonstrated that circRNAs can act as sponges to bind to miRNAs, regulate transcription, or affect gene expression, and we believe that there might be other functions remaining to be revealed. Recently, a circRNA database has been constructed (http://circnet.mbc.nctu.edu.tw/) [66]. This database provides tissue-specific circRNA expression profiles and circRNA-miRNA-gene regulatory networks [66].

As is known to all, miRNAs regulate cardiac function through regulating the development, differentiation, proliferation, and apoptosis of cells during the progression of diseases [41, 67, 68]. Besides, abnormal expression of miRNA may induce cardiac fibrosis through targeting TGF-*β*, including miR-378, miR-122, miR-29, and miR-26 [69]. Moreover, one miRNA can have many target mRNA [42, 70], while one mRNA can be managed by several miRNAs [71]. In this review, we summarized recent studies on the interaction of circRNAs with others and the relationships between circRNAs and cardiovascular diseases, hinting that circRNAs play special regulating roles in the initiation and progression of related diseases. We noted two circRNAs, HRCR and Cdr1as, which could function as miRNA sponges in the cardiovascular diseases, indicating that some circRNAs may act as upstream regulators of miRNAs. As roles of many miRNAs in different cardiovascular diseases have been verified [72–75] and several circRNAs have been detected in the myocardium of humans and mice [43], we think more studies can be done in the future to figure out the link of circRNAs and miRNAs. Therefore, we speculate that circRNAs may serve as diagnostic or prognostic biomarkers of disease and have potential therapeutic values. However, more studies have to be done before we fully understand the biological and molecular mechanisms of circRNAs in the development of cardiovascular diseases. Perhaps in the future, circRNAs may have great potential in clinical diagnosis and treatment of disease. With the development of new technologies, more circRNAs will be detected as well as their new functions.

## Figures and Tables

**Table 1:** An overview of circular RNAs in various cardiovascular diseases.

	Diseases type	CircRNA	Targets	Effect on diseases	References
a	Heart failure and pathological hypertrophy	HRCR	miR-223 ARC	Suppress	[40]
b	Myocardial Infarction	Cdr1as(ciRS-7)	miR-7 SP1, PARP	Induce	[41]
c	Cardiac senescence	circ-Foxo3	ID1, E2F1, FAK, HIF1a	Induce	[42]
d	Atherosclerosis	cANRIL	The INK4/ARF locus	Regulate	[43]

a: HRCR can act as miRNA sponges and bind to miR-223. ARC, a kind of protein, is the downstream target of miR-223, so that HRCR can enhance ARC's protective role as blocking the progression of cardiac pathological hypertrophy and heart failure through inhibiting miR-223 activity. b: Cdr1as can function as miR-7 sponges. SP1 and PARP are miR-7 target genes and can inhibit miR-7-induced protective role during MI development. c: Circ-Foxo3 can hinder transcription factors' (ID1, E2F1, FAK, and HIF1a) transfer into nucleus so as to repress their antiageing effect. d: CANRIL is an antisense transcript from the INK4/ARF locus resulting from alternative ANRIL transcription and splicing, while cANRIL can also influence the PcG-mediated INK4/ARF silencing. As SNPs on chromosome 9p21 near the INK4/ARF locus can control INK4/ARF expression, which is correlated to atherosclerosis susceptibility, cANRIL can have a regulatory role here.
